# The Effect of Polyphenols on Kidney Disease: Targeting Mitochondria

**DOI:** 10.3390/nu14153115

**Published:** 2022-07-29

**Authors:** Fatemeh Ashkar, Khushwant S. Bhullar, Jianping Wu

**Affiliations:** Department of Agricultural Food and Nutritional Science, University of Alberta, Edmonton, AB T6G 2R3, Canada; fashkar@ualberta.ca (F.A.); bhullar@ualberta.ca (K.S.B.)

**Keywords:** kidney, mitochondrial function, polyphenols, acute and chronic renal diseases

## Abstract

Mitochondrial function, including oxidative phosphorylation (OXPHOS), mitochondrial biogenesis, and mitochondria dynamics, are essential for the maintenance of renal health. Through modulation of mitochondrial function, the kidneys are able to sustain or recover acute kidney injury (AKI), chronic kidney disease (CKD), nephrotoxicity, nephropathy, and ischemia perfusion. Therapeutic improvement in mitochondrial function in the kidneys is related to the regulation of adenosine triphosphate (ATP) production, free radicals scavenging, decline in apoptosis, and inflammation. Dietary antioxidants, notably polyphenols present in fruits, vegetables, and plants, have attracted attention as effective dietary and pharmacological interventions. Considerable evidence shows that polyphenols protect against mitochondrial damage in different experimental models of kidney disease. Mechanistically, polyphenols regulate the mitochondrial redox status, apoptosis, and multiple intercellular signaling pathways. Therefore, this review attempts to focus on the role of polyphenols in the prevention or treatment of kidney disease and explore the molecular mechanisms associated with their pharmacological activity.

## 1. Introduction

**Figure 1:**
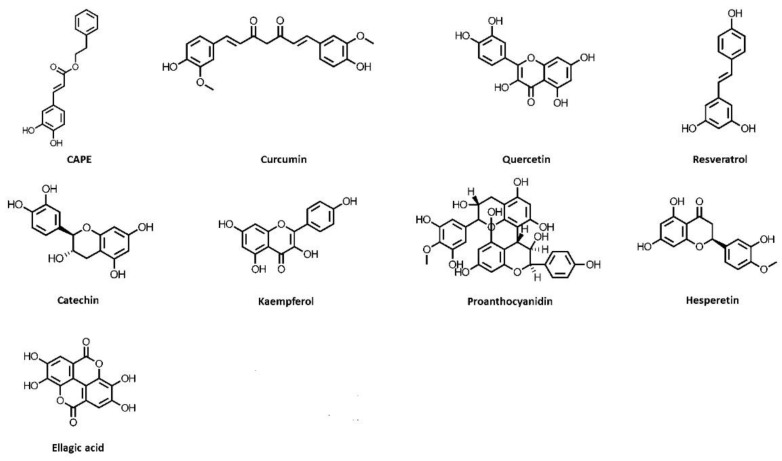
Chemical structures of polyphenols, which exhibit kidney-protective activities. CAPE, caffeic acid phenethyl ester.

Kidneys are one of the most energy-demanding organs and play a vital physiological role in the maintenance of salt and water homeostasis [1]. Kidneys receive approximately 25% of the cardiac output and are responsible for the regulation of blood pressure and continuous blood filtration [2]. Physiologically, kidneys consume about 7% of the total oxygen available for overall human function, indicating a significant role of mitochondria in their physiology [2]. Mitochondria are abundant in metabolically active organs, including kidneys, especially in the renal tubule cells [3,4]. Indeed, the kidney is a metabolically active organ containing more mitochondria per weight than any other human organ [5,6]. Acute and chronic kidney diseases, such as renal ischemia, toxicity, and acute injury, include underlying mitochondrial dysfunction [7,8,9]. Research has established links between both acute and chronic kidney diseases with impaired mitochondrial biogenesis, OXPHOS, and mitochondria mitophagy [10]. The mitochondrial dysfunction in kidneys is also linked to inflammation, apoptosis, and tissue injury, thus, contributing to mortality and morbidity rates [11]. Studies have shown that dietary patterns and dietetic components could modulate renal function and disease [12,13]. A diet rich in plants, vegetables, and fruits is related to a lower incidence of chronic diseases, such as cardiovascular disease, cancers, type 2 diabetes, and kidney disease(s) [14,15]. These biological functionalities are associated with the presence of active antioxidants, particularly polyphenols [15]. ‘Polyphenol’ is not a strict chemical term and is used to refer to flavonoids, tannins, and phenolic acids and their various chemically modified or polymerized derivatives [16]. Over the last two decades, multiple polyphenols have attracted attention as nephro-protective agents, particularly owing to their ability to maintain oxidative homeostasis and activate cytoprotective signaling in vivo (Figure 1) [17]. Recent studies have shown the therapeutic effects of bioactive compounds and their beneficial health effects; however, little effort has been put into summarizing the impact of polyphenol interventions on mitochondrial dysfunction in various renal diseases [12,18,19]. This literature review attempts to focus on the role of polyphenols in the prevention and/or treatment of kidney disease and explore the cellular mechanisms associated with their pharmacological activity. We mainly focus on preclinical studies, both cellular and animal, that displayed the ability of polyphenols to decrease physiological complications and enhance mitochondrial function.

## 2. Bioavailability of Polyphenols

Recent studies have reinforced the health-promoting evidence of polyphenols based on diverse experimental models [20,21]. However, their chief problems are their low bioavailability and rapid metabolism [22]. Therefore, the bioavailability of polyphenols has been considered a significant limitation for their clinical evaluation and translations.

After polyphenol administration, oxidation, reduction, hydrolysis, and conjugation cause the production of different water-soluble conjugate metabolites, which can pass the enteric barrier for further distribution to organs [20,23]. These processes are mediated by lactase phlorizin hydrolase (LPH) and cytosolic β-glucosidase (CBG) [24]. Multidrug-resistance-associated proteins (MRP-1 and MRP-2) also play essential roles in polyphenol bioavailability and tissue accumulation [25]. During intestinal transit, MRP-2 on the apical surface of cells transports intracellular polyphenols to the lumen of the intestine. MRP-1, located in the vascular pole of enterocytes, promotes polyphenol passage from the enterocyte into the bloodstream [24]. MRP-3 and the glucose transporter 2 (GLUT2) efflux polyphenol metabolites from the enterocytes basolateral membrane to the portal circulation and reach the liver [24]. It is reported that small intestines can only absorb about 5–10% of the total polyphenol intake after deglycosylation [26]. About 90–95% of unmodified polyphenols and the conjugated forms pass through the intestinal tract to the large intestine for gut microbiota action. Gut microbiota can produce various metabolites to exert physiological impacts [27].

Despite the relatively few studies demonstrating lower mitochondrial uptake of polyphenols, their lipophilicity and pKa make them more suitable for mitochondrial enrichment [28]. A recent study showed that polyphenols were more bioavailable and could reach mitochondrial sites of action than previously assumed [29]. The pH value of cells affects the diffusion of polyphenols. Polyphenols are neutral phenols and form phenolate anions in the cytosol [29,30]. Their lipophilicity determines their ability to cross cell membranes and inner- and outer-mitochondrial membranes. Due to their pKa values close to the cytosol’s and mitochondria’s pH and distribution coefficients, many polyphenols can reach the mitochondrial matrix and release a proton in a relatively basic environment. [29]. At that time, phenolate anions move back down the electrochemical gradient to the relatively acidic intermembrane space. Protons are then transported from the inner-mitochondrial membrane to the matrix to regulate the electrochemical gradient (ΔΨm) [29,30]. In general, studies have shown that polyphenols are bioavailable and their metabolism via different mechanisms is responsible for their biological activities [31,32].

## 3. Mitochondria and Kidneys

### 3.1. Oxidative Phosphorylation (OXPHOS) System

Mitochondria are the central site for over 90% ATP production in cells [33,34]. ΔΨm in mitochondria is critical for mitochondrial function and is widely used as an indicator for mitochondrial function and oxidative stress [35]. The overproduction of reactive oxygen species (ROS), primarily superoxide anion (O2^·−^), during the transfer of electrons to oxygen, and a deficiency in antioxidant enzymes, such as superoxide dismutase (SOD) and glutathione (GSH) [36], leads to oxidative stress, mitochondrial dysfunction, and apoptosis [37]. Because mitochondrial ROS can inhibit multiple signaling pathways and prevent redox-dependent proteins’ proper function and activity, it is reported that mitochondrial ROS could be detrimental to cell survival and the health of a kidney cell [38]. ROS are produced in both the renal cortex and medulla, resulting in altering renal blood flow, inflammation, fibrotic changes, and proteinuria [39].

### 3.2. Mitochondrial Biogenesis

Mitochondrial biogenesis is an intricate and adaptive cellular response process [40]. It requires coordinated transcription and replication of mitochondrial DNA accompanied by the synthesis and import of proteins [5]. The mitochondrial biogenesis is regulated by the proliferator-activated receptor-gamma coactivator-1α (PGC-1α) family of transcriptional coactivators [12]. Mitochondrial biogenesis, respiration, fatty acid β-oxidation, and OXPHOS are all controlled by the interaction of PGC1-α with different transcription factors, such as nuclear respiratory factors 1 and 2 (Nrf1/2) and peroxisome proliferator-activated receptors (PPARα) [38]. The PGC-1α transcriptional coactivator is highly expressed in the proximal tubules of the kidney and plays a critical role in tubular homeostasis [11]. AMP-activated protein kinase (AMPK) and family of NAD^+^-dependent deacetylases known as Sirtuins (SIRT1–7), including SIRT1, are essential modulators of energy metabolism. AMPK with phosphorylation and SIRT1 through deacetylation can positively regulate PGC-1α [41,42,43]. Stimulation of PGC-1α through deacetylation or phosphorylation can stimulate the pathway followed by activation of nuclear transcription series factors, such as Nrf1, Nrf2, and transcription factor A mitochondria (TFAM) expression, consequently leading to mitochondria DNA (mtDNA) transcription and replication [44]. Moreover, PGC-1α activation improves the nicotinamide adenine dinucleotide (NAD^+^) biosynthesis, a key molecule critical for oxidative metabolism and cell protection [11]. It is reported that transgenic expression of PGC-1α leads to increased mitochondrial content and expression of mitochondrial genes. Conversely, loss of PGC-1α results in reducing the mitochondrial genes expression and causes mitochondrial dysfunction in mice [38]. There has been widespread evidence of reduced mitochondrial biogenesis as well as low PGC-1α levels in AKI and CKD [45]. Further, the Nrf2 antioxidant pathway was established to cope with CKD-induced oxidative stress in renal cells. Nrf2 is bound to its repressor under normal physiological conditions; under oxidative stress, Nrf2 is rapidly dissociated and translocated to the nucleus, encoding the antioxidant enzyme gene [46]. On the other hand, ROS, oxidative stress, and inflammation suppress the antioxidant potential of renal cells by suppressing the expression of Nrf2 [47]. Cellular homeostasis is integrated with function of mitochondria and biogenesis. It leads to metabolic syndrome, neurodegenerative diseases, and cancer if the intracellular pathway is malfunctioned [44]. According to the broad involvement of PGC-1α and Nrf1/2 as the important factors of mitochondria biogenesis, they can serve as a vital pharmacological target in metabolic diseases.

### 3.3. Mitochondrial Dynamics

To maintain cellular homeostasis and mitochondrial function, mitochondrial dynamics, such as division, fusion, and movement, are indispensable [48,49,50]. There are also fission proteins regulating mitochondrial dynamics, including mitochondrial fission 1 (Fis1), fusion proteins, and optical atrophy (OPA1) [7,51]. For the optimal function of mitochondria, there must be a balance between fission and fusion events, since imbalanced mitochondrial dynamics will eventually result in diseases, such as insulin resistance and type 2 diabetes, hypertension, cardiovascular diseases, and obesity [11,38,52]. Further, kidney disease and impairment are related to an increased mitochondrial fragmentation [53]. These findings suggest that a balanced mitochondrial fission and fusion is necessary for optimal mitochondrial function in kidney cells.

### 3.4. Mitophagy

Mitophagy is the autophagy of accumulated dysfunctional mitochondria modulated by PTEN-induced putative kinase 1 (PINK1)-parkin RBR E3 ubiquitin protein ligase (PARK2) pathways (ubiquitin-dependent mechanism) and B-cell lymphoma 2 (Bcl2) interacting protein 3 (ubiquitin-independent mechanism) [3,54,55,56]. There is an association between disturbed mitophagy and kidney diseases, such as acute kidney injury, diabetic nephropathy, and glomerulosclerosis [11]. In PINK1 and/or PARK2 knockout models, ROS production, inflammation, mitochondrial fragmentation, and cell apoptosis were enhanced in kidney cells, resulting in severe kidney injury. This suggests that PINK1 and PARK2 pathways act as a protective mechanism in AKI to maintain renal tubular integrity and kidney function [57].

## 4. Kidney and Mitochondria

Chronic and acute kidney injuries are linked with the production of ROS and reactive nitrogen species (RNS) [11]. Oxidative stress in AKI results from sepsis, ischemia-reperfusion injury, exposure to nephrotoxic reagents, and diabetic nephropathy. It was revealed that a balance between fission and fusion tended toward fission, contributing to mitochondrial fragmentation in AKI [58]. As a consequence, fragmentation could be related to the release of apoptotic factors, such as cytochrome *C*, activation of caspase, and apoptosis [53]. Additionally, AKI in cell and mouse models showed a decrease in mitophagy, ROS production, inflammation, and increase in mitochondrial damage [59]. Renal fibrosis and, consequently, CKD usually results from repeated or severe AKI [60,61,62]. Further, CKD may arise from environmental exposure to metal, pesticides, and infectious agents, decreased glomerular filtration rate, and higher urinary albumin excretion [63,64]. Enhanced fragmentation of mitochondria in kidney tubules, the reduced mitochondrial biogenesis, loss of mitochondria membrane potential (MMP), drop in ATP generation, and overproduction of mitochondrial ROS were reported in CKD [38,65]. Thus, CKD and AKI might perturb mitochondria biogenesis, dynamics, and mitophagy clearance. The conditions are all likely to lead to an accumulation of inflammatory cytokines, release of pro-apoptotic factors, and tissue damage [11].

An ischemic/reperfusion (I/R)-injury-induced AKI is a cellular injury that is triggered by a pathological condition that results in blood returning to tissues that have been ischemic [66]. I/R contributes to kidney dysfunction and AKI [67]. It is accompanied by inflammation, ROS and cytokine generation, lipid peroxidation, changes in mitochondrial function, and mitochondria injury [68,69]. I/R could increase the protein levels of pro-inflammatory factors, including tumor necrosis factor α (TNF-α), interleukin 1β (IL-1β), and interleukin 6 (IL-6), and levels of the ROS and malondialdehyde (MDA), while decreasing SOD and GSH [70]. In mitochondria, cytochrome oxidase (complex IV) is able to catalyze electron transfer from cytochrome *C* to oxygen to produce a proton gradient for ATP synthesis [71]. ROS and lipid peroxidation products effectively inhibit mitochondrial complex IV activity [36,72], thus, influencing the electron flow across the electron transport chain and ATP production [73]. As a result of lipid peroxidation, different pathways lead to apoptosis and autophagy [74]. In another study, the Nrf2/heme oxygenase-1 (HO-1) signaling pathway decreased renal I/R injury by mediating oxidative stress [75]. Ca^2+^ at physiological concentrations is an essential regulator of mitochondrial energy metabolism [76]. Ca^2+^ influx into the mitochondria is a noteworthy factor in triggering mitochondrial ROS production [77]. Overproduction of ROS might result from increased mitochondrial Ca^2+^ accumulation, leading to inhibition of electron transport and/or increase in the enzymes responsible for ROS generation [78]. The mitochondrial Ca^2+^ load reduces the transmembrane potential and opens the mitochondrial permeability transition pore (MPT), damaging mitochondria and mitochondrial respiratory chains and subsequent ROS surge [79]. On the other hand, it was found that ischemic injury decreased the OXPHOS and Ca^2+^ uptake in kidney mitochondria, which could impact mitochondrial metabolism [69]. These studies demonstrated that I/R-induced inflammation, oxidative stress, and apoptosis might be related to kidney mitochondria. Acute kidney injury resulting from nephrotoxicity could damage mitochondria and, consequently, impair renal functions [80]. 

Cadmium is a toxic heavy metal, which has extensive nephrotoxic impact [81]. The expression of PGC-1α, Nrf1, SIRT1, and TFAM involved in mitochondrial biogenesis were impaired in cadmium-induced nephrotoxicity [82]. Nephrotoxicity caused mitochondrial fission by inhibiting mitochondrial membrane fusion and activating mitophagy mediated by the PINK/Parkin pathway [83]. Cadmium-induced renal impairment might alter tissue redox status by increasing lipid peroxidation products, such as MDA and nitrite oxide (NO), and decreasing SOD and catalase (CAT) enzymes in the kidneys [84]. This leads to disruption in mitochondria function, mitochondrial membrane potential, and eventually, renal hemostasis [82,85,86].

The antibiotic gentamycin is widely used to treat bacterial infections [87]. Nephrotoxicity caused by gentamycin also triggers ROS production in mitochondria, stimulating the opening of the MPT pore [88]. Thus, the MPT pore opening triggers the release of cytochrome *C* into cytosol which leads to swelling of mitochondria, activation of caspase cascade, and finally culminates to apoptosis [89]. In addition, the Bcl-2/Bcl-2-associated X (Bax) ratio, which is a vital factor to control cell apoptosis, decreased in the kidney following nephrotoxicity [90].

Anticancer drugs, such as cisplatin, cause DNA crosslinking and apoptosis [91]. Likewise, cisplatin-induced nephrotoxicity elevated protein oxidation and lipid peroxidation in the kidney mitochondria of rats, resulting from increasing ROS production or decreasing antioxidant status [92]. After cisplatin administration, the levels of the lipid peroxidation end-product MDA were significantly increased along with GSH and SOD depletion in rats [93]. The enhanced lipid peroxidation in mitochondria might cause decreased mitochondrial membrane fluidity, an increase in the distribution of negative surface charge, and an altered ionic membrane permeability [94]. Cisplatin triggers signaling cascades, such as p53, MAP kinase (MAPK), and nuclear factor kappa B (NF-κB), by ROS formation [95]. Further, cisplatin released pro-inflammatory cytokines, for instance, interleukin 12 (IL-12), TNF-α, and IL-1β to induce kidney damages [96]. Therefore, cisplatin was able to damage the kidney by generating oxidative stress, inflammation, DNA damage, apoptosis, and mitochondrial dysfunction [97].

Cyclosporine A is an immunosuppressive drug used to treat autoimmune diseases and to prevent organ rejection [98]. Studies indicated that cyclosporine A could cause acute and chronic nephrotoxicity by inhibiting mitochondrial respiration and decreasing ATP production in vivo and in vitro [99,100,101,102]. Cyclosporine A might suppress mitochondria biogenesis to induce nephrotoxicity [103]. Human kidney proximal tubule epithelial cells treated with cyclosporine A showed increased mitochondrial dysfunction and cellular death induced by H_2_O_2_. ROS production during H_2_O_2_ injury could activate the p53 pathway. In addition to binding DNA, activated p53 could accumulate in the mitochondrial matrix and trigger necrotic cell death by opening the MPT pore [104].

Doxorubicin, an anticancer agent, is widely used in the treatment of leukemia, breast cancer, and solid tumors [105]. Similar to other nephrotoxic drugs, there was an association between doxorubicin exposure and declining antioxidant parameters, such as glutathione peroxidase (GPx), SOD, and CAT, as well as SIRT1 activity [106,107]. Research showed that doxorubicin elevated thiobarbituric acid reactants (TBARS) and MDA, an indicator of oxidative damage [108]. NF-κB activation plays a critical role in the pathogenesis of doxorubicin-induced renal inflammation [109]. According to this, NF-κB was responsible for inflammatory reactions by mediating TNF-α, IL-1β, and IL-6 expressions in rats treated with doxorubicin [110]. The formation of superoxide radical(s) by doxorubicin exposure led to apoptosis [111,112]. Further, doxorubicin-treated animals showed cell death and apoptosis characterized by upregulation of Bax, down-regulation of Bcl2, increased mitochondrial permeability, and activation of caspase-3 in kidneys [106].

Diabetic nephropathy, a complication of microvascular in diabetes, could cause renal disease [113]. Redox changes are caused by persistent hyperglycemia and the accumulation of advanced glycation end products (AGEs) [114]. The resulting chronic inflammatory response leads to aberrant redox changes, albuminuria, proteinuria, glomerulosclerosis, and tubule-interstitial fibrosis [115]. Complications associated with diabetes are caused by ROS production, can damage mitochondrial DNA, and induce cell dysfunction [116,117]. These changes in renal cells, including glomerular endothelial cells, mesangial cells, and renal epithelial cells, disrupt ATP synthesis, cause intracellular calcium imbalances, and contribute to apoptosis and necrosis [118]. Diabetic rats’ kidney tissues showed higher levels of ROS, MDA, TNF-α, IL-6, and NF-κB p65 [119]. Apoptosis was also observed with higher Bax protein and cleaved caspase-3 levels, increased cytochrome *c* cytoplasmic levels, and Bcl2 down-regulation. In addition, the kidneys of diabetic rats revealed a significant decrease in the mRNA levels and nuclear levels of Nrf2, with a reduction in SOD mRNA levels and SOD and GSH protein levels. This disruption in cellular viability and oxidative homeostasis was possibly backed by hyperglycemia-induced ROS surge and depleted Nrf2 pool [120]. In diabetic nephropathy, the oxidative stress might increase GSH degradation or lower innate GSH synthesis. Moreover, ROS also lower the enzymatic activities of SOD and CAT [121]. Further, free radicals induced during diabetic nephropathy lowered the activity of AMPK and SIRT1, the critical regulators of PGC1α activity and energy metabolism of mitochondria [122]. The injury of the podocyte cells that cover the outer surfaces of glomerular capillaries, related to Nrf1 and mitochondrial dysfunction, contributed to diabetic kidney disease [123]. Studies have also shown that mitochondrial damage contributed to chronic and acute kidney injury as a result of a reduction in mitochondrial DNA, mitochondrial membrane potential, and ATP production along with increase in inflammation, and apoptosis [65].

## 5. Antioxidants and Kidney Diseases

### 5.1. Caffeic Acid Phenethyl Ester

Caffeic acid phenethyl ester (CAPE) is a natural phenolic compound possessing anti-inflammatory, antioxidant, and immunomodulatory effects [124]. CAPE exhibits a strong antioxidant potential by scavenging free radicals and facilitating oxidative homeostasis [125]. Further, CAPE improved OXPHOS of mitochondria through complex-I-dependent substrate(s) glutamate/malate [69]. It was later shown that CAPE pre-treatment protected complex II (SDH) activity and inhibited ROS formation at the Complex II F [68]. CAPE reduced Fe^3+^ (oxidized form of cytochrome *C*) into Fe^2+^, inhibiting the release of cytochrome *C* to cytosol and apoptosis. This protection decreased MDA and xanthine oxidase (XO), while increasing antioxidant enzyme GSH [68]. Therefore, CAPE inhibited lipid peroxidation in renal tissues [126]. Further, CAPE pre-treatment ameliorated mitochondrial swelling and dissipation of membrane potential following renal toxicity by cadmium [127]. Özeren et al. [128] showed that CAPE prevented kidney ischemia/reperfusion injury by inhibiting lipid peroxidation and improving mitochondrial Ca^2+^ uptake, resulting in improved mitochondrial energy metabolism [69]. Furthermore, CAPE treatment also boosted levels of NO from endothelial cells, thus preventing the pathological damage in ischemia [129]. Consequently, CAPE increased mitochondrial function to uptake calcium and boost OXPHOS [69,129]. Lastly, CAPE was able to lower oxidative stress, increase antioxidant enzyme activity and GSH content, and inhibit MPT pore opening, resulting in improved renal health [130]. Additionally, CAPE blocked ROS production and augmented the activity of antioxidant enzymes, such as SOD and CAT [126]. Since CAPE exhibits potent antioxidant, anti-inflammatory, and mitochondrial protective effects in kidney cells and tissues, this promotes CAPE as a promising new therapeutic agent that has the potential to protect the kidney from damage [126].

### 5.2. Curcumin

Curcumin is a natural polyphenol product derived from the rhizome of the *Curcuma longa,* exerting anti-inflammatory, antioxidative, anti-tumor, and anti-fibrotic effects [131]. The presence of conjugated double bonds in the curcumin structure allows it to donate an electron and scavenge ROS [132]. Curcumin has shown a protective effect in kidney damage models via its antioxidant activity, leading to the preservation of mitochondrial function [133]. Further, curcumin prevented mitochondrial dysfunction by protecting the mitochondrial respiratory complexes [134]. Some drugs, including gentamycin, reduce the activity of complexes I, II, and IV [134]. The complexes I and IV concentration and activities were recovered through curcumin treatment [134]. Consequently, the phosphorylation efficiency (Adenosine di-phosphate (ADP)/Oxygen) ratio in mitochondria oxidizing malate/glutamate and uncoupled respiration was recovered and redox homeostasis was maintained to prevent mitochondrial dysfunction. Curcumin suppresses TNF-α-mediated NF-κB activity in the development of chronic renal failure and inflammation [135,136]. Further, curcumin reduced interferon gamma (IFNγ) expression, but increased IL-10 levels in the renal ischemia/reperfusion model [137]. 

Curcumin also exhibited protective impacts against various nephrotoxic agents, such as cisplatin, gentamicin, and cadmium [138]. Particularly, curcumin treatment increased the PGC-1α levels and TFAM expression in nephrotoxcity-induced AKI [139,140]. Curcumin also protected the kidneys from oxidative stress in cisplatin-induced nephrotoxicity [141]. For example, curcumin attenuated oxidative stress and lipid peroxidation by scavenging ROS, restoring manganese superoxide dismutase (MnSOD) activity, enhancing glutathione s transferase (GST) activity, and modulating the GSH levels in kidney mitochondria [142]. Mechanistically, curcumin protected against cisplatin-induced oxidative damage by activating transcription factor EB (TFEB), leading to the regulation of autophagy and decreased levels of ROS after elimination of damaged mitochondria [143]. Moreover, curcumin was also able to restore the imbalance of mitochondrial dynamics in cisplatin nephrotoxicity through attenuation of Fis1 levels and restoring OPA1 levels [144]. Curcumin significantly regulated SIRT3, leading to mitochondrial integrity, a decrease in mitochondrial fission, and improved mitochondrial fusion. SIRT3 upregulation by curcumin also reduced dynamin-related protein 1 (DRP1) levels and prevented depolarization of the mitochondrial membrane in nephrotoxicity with cisplatin [142,145]. Further, curcumin treatment showed a higher number of normal-structure mitochondria and lower swollen mitochondria in gentamicin-induced kidney damage, owing to its ability to recover oxygen consumption of mitochondria [134]. Additionally, curcumin also ameliorated the MPT pore opening and protected them from the detrimental effects by preserving mitochondrial integrity [134]. Curcumin also showed protective effects in rats with a renal interstitial fibrosis model. In this study, curcumin inhibited the PI3K/Akt mammalian target of the rapamycin (mTOR) signaling pathway activation and upregulated essential proteins, mediating autophagosome formation. This led to suppressing the inflammatory response and mitochondrial dysfunction development [131]. Furthermore, the ability of curcumin to boost mitochondrial biogenesis warrants its exploration and use for renal disease [146].

### 5.3. Quercetin

Quercetin, a natural flavonoid abundant in fruits, vegetables, and leaves, is a potent antioxidant, which alleviates cell senescence by reducing oxidative stress [107,147]. Quercetin alleviates oxidative stress, prevents kidney damage, and inhibits renal inflammation in animal models of diabetic nephropathy [148]. Further, quercetin treatment prevented structural and functional damage of renal tissue and suppressed oxidative stress in the rats with tubulointerstitial necrosis and cadmium nephrotoxicity [149]. Recently, it was found that quercetin had chemo-protective and anti-apoptotic effects as a result of elevated expression of p53, p21, and p27 and lowered Bax expression in vitro [150]. Quercetin chelated metal ions, such as iron and copper, which were able to scavenge free radicals in in vitro experiments [151]. Quercetin also suppressed NF-κB, lipid peroxidation, and expression of pro-inflammatory matrix metalloproteases, whereas it might elevate nitric oxide levels and non-enzymatic antioxidant capacity of plasma [107]. Quercetin also ameliorated nephrectomy-induced oxidative stress by increasing GPx and decreasing MDA levels in rats [46,152]. In addition, quercetin restored mitochondrial function and protected against DNA double-strand breaks after doxorubicin treatment in H9c2 cells [153]. It was shown that quercetin could increase the expression of Nrf2 in the nucleus to enhance the encoding of antioxidant enzymes and gene expression of HO-1 in rats with CKD [46]. In renal interstitial fibrosis, quercetin significantly enhanced mitophagy by activating SIRT1 and inducing the PINK1-Parkin signaling pathway [153]. Moreover, a reduction in systolic blood pressure was associated with a reduction in epithelial Na^+^ channel (ENaCs) expression in the kidneys of hypertensive Dahl salt-sensitive rats treated with quercetin [154,155]. Based on the studies, quercetin can be considered a polyphenol with the ability to lower oxidative stress and apoptosis, while improving mitochondria mitophagy and biogenesis in the kidney.

### 5.4. Resveratrol 

Resveratrol is a natural stilbenoid polyphenol found in grapes, blueberries, and peanuts [156]. It exhibits anti-inflammatory, anti-cancer, and anti-aging effects, both in cells and in animals [157]. Further, resveratrol has potential in the treatment of kidney diseases to improve overall health [34]. Studies observed that resveratrol enhanced the NADH entry into electron transport, thus, increasing the NAD^+^-NADH ratio, which might influence SIRT1 activity [72,158]. There is ample evidence indicating that resveratrol increased all SIRT1 target proteins, which were critical to mitochondrial function and oxidative stress reduction in kidneys [159]. Resveratrol-induced SIRT1 activity triggered a decrease in fibrosis, mesangial expansion, oxidative stress, and inflammatory cytokine levels, resulting in improved kidney function [160,161]. In the kidneys of SIRT1 KO db/db mice, the expression of pro-inflammatory factors mediated by NF-κB and signal transducer and activator of transcription 3 (STAT3) rose dramatically, supporting resveratrol-induced SIRT1’s crucial role in kidney inflammation [162]. Likewise, resveratrol protected against diabetic kidney disease in db/db mice with type 2 diabetes via an AMPK/SIRT1-independent mechanism [163]. The treatment of db/db mice with 20 mg resveratrol/kg/day for 12 weeks led to a reduction in kidney damage and modification of renal diabetes phenotypes [164]. A recent study revealed that resveratrol was essential in restoring mitochondrial function and biogenesis via SIRT1/PGC-1α activation in kidneys of diabetic mice [165]. It was shown that activation of SIRT1-dependent pathways by resveratrol attenuated kidney injury by upregulation of mitochondrial biogenesis factors [72]. Further, in chickens that were treated with resveratrol, Nrf2 signaling was activated to reverse renal oxidative damage caused by cadmium injury and activate downstream phase II detoxification factors, such as HO-1, NAD(P)H dehydrogenase quinone 1 (NQO1), and GSTs [82]. Likewise, Kim et al. proved that a reduction in oxidative stress through Nrf2 activation ameliorated renal function, proteinuria, and pathological changes in aging mice [157]. Alternatively, resveratrol treatment prevented a decrease in the activity of complex II and complex IV following hemorrhagic shock, which decreased ROS production and damage in a rat model of kidney disease [72]. Additionally, Hui et al. showed that resveratrol treatment raised MMP and activities of complex I and III; therefore, the production of ATP improved and reduced the generation of ROS in a rat model of CKD [34]. Further, Zhang et al. showed that resveratrol reversed mitochondrial injury, diminished the autophagic vacuole number, and ameliorated mitochondrial fission in chicken kidney [82]. In addition, by improving mitochondrial elongation, resveratrol facilitated autophagy, suppressed Parkin and PINK1 phosphorylation, and degraded mitochondria that were removed [82]. Generally, these studies suggested that treating renal injuries with resveratrol might attenuate nephrotoxicity, I/R, oxidative stress, and apoptosis, while increasing antioxidant enzyme activities. In addition, resveratrol treatment might affect mitochondrial biogenesis and dynamics in kidney diseases to ameliorate mitochondrial dysfunction and metabolic stress.

### 5.5. Catechin 

Catechin, as a part of the flavonoids family, is present in plants, fruits, teas, red wine, and cacao [166]. In addition to having antioxidant properties, it also exhibits potent anti-inflammatory properties [167]. Catechin protects the kidneys by scavenging free radicals, inhibiting intracellular ROS, chelating redox-active metals, and enhancing antioxidant defense mechanisms [168,169]. In addition, catechin had the potential to prevent MMP loss and apoptosis by restoring the activity of mitochondrial complex I and ATP synthesis [170]. In SK-N-MC cells, catechin boosted the expression of anti-apoptotic protein Bcl-2 and inhibited the expression of apoptotic protein Bax [171,172].

Epigallocatechin gallate (EGCG) is a catechin esterified with gallic acid [173]. It is the major polyphenol in green tea with antioxidant activity in reducing mitochondrial oxidative stress [174,175]. It was found that EGCG restored mitochondrial electron transport chain function to normal in mouse kidney with cisplatin-induced damage [176]. Further, EGCG protected against renal injury caused by cisplatin through favoring mitochondrial antioxidant enzymes, such as MnSOD and GPx, and enhancing the anti-inflammatory effect [177]. Further, EGCG treatment significantly reduced DNA damage caused by p65 and P53 and modulated NF-κB nuclear accumulation in cisplatin nephrotoxicity [176]. In the rat model of obstructive nephropathy, treatment with EGCG inhibited NF-κB activation, while improving the phosphorylated IkappaB (IκB) protein and inducing Nrf2 nuclear translocation [177]. EGCG induced GST, GPx, and HO-1 expression, where they were able to eliminate or inactivate ROS and oxidative stress; thus, it could suppress oxidative stress and acute renal injury [178,179]. In a mouse model of nephrotoxicity, EGCG modulated the receptor Bax and Bcl-2 that attenuated cisplatin-induced apoptosis [180]. Thus, EGCG-induced modulation of NF-κB and Nrf2 is a critical element for oxidative stress and inflammation alleviation in acute kidney damage [177,181]. Furthermore, green tea polyphenols (polyphenol + catechin + EGCG) protected the rat kidneys from the oxidative damage caused by a high-fat diet via an SIRT3/MnSOD pathway mediated by PPARα [182]. It was suggested that green tea polyphenols increased PGC1-α and TFAM axis, mitochondria DNA, OXPHOS proteins, and SIRT1 activity related to a reduction in kidney injury and improvement in renal function after cyclosporine treatment of rats [103]. Eventually, EGCG and catechin had the ability to enhance mitochondria function by impacting biogenesis, dynamics, and OXPHOS to prevent or treat kidney diseases.

### 5.6. Kaempferol

Kaempferol, a natural flavonoid, is found in tea, vegetables, and fruits, such as broccoli, grapes, kale, tomatoes, and citrus fruits [183,184]. Kaempferol has antioxidant, anti-cancer and anti-inflammatory effects [97]. It was reported that kaempferol caused a significant decline in MDA levels, an indicator of oxidative stress, cytotoxicity, and renal damage in calcineurin inhibitor-induced renal injury and CKD [185]. In addition, kaempferol could lower lipid peroxidation and improve antioxidant defense activity [186]. Tumor necrosis-factor-receptor-associated factor 6 (TRAF6), a transcription factor upstream of NF-κB, is downregulated by kaempferol, reducing renal inflammation and fibrosis in renal tubular epithelial cells [187]. It was shown that pre-treatment of kaempferol reduced pro-inflammatory cytokine release, such as IL-12 and TNF-α, and regulated NF-κB levels by hindering the IkappaB kinase (IKK) phosphorylation and IκBα degradation; thus, it ameliorated the cisplatin-mediated inflammation in mouse kidney proximal tubule epithelial (TKPTS) cells [97]. Furthermore, kaempferol inhibited the p38, ERK, and c-Jun N-terminal kinase (JNK) activation, while augmenting Coenzyme Q (CoQ) biosynthesis and content [97]. Treatment with kaempferol increased GSH and SOD2, while reducing TNF-α and IL-6 in the kidneys of doxorubicin-treated rats [106]. Moreover, treatment and pre-treatment with kaempferol in rats increased nuclear accumulation of Nrf2, which was necessary for mitochondrial biogenesis, in contrast to the cisplatin- and doxorubicin-treated animals [106,180]. In addition, the protective effects of kaemepferol against streptozotocin-induced diabetic nephropathy could be attributed to its potent antioxidant effect, mediated by upregulation and activation of Nrf2 [188]. Overall, kaempferol can be a potential therapeutic used in treatment, preventing kidney mitochondria injury because it has anti-inflammatory and antioxidative properties.

### 5.7. Grape Seed Proanthocyanidin

Other plant polyphenols, such as grape seed proanthocyanidin extracts (GSPE), have strong therapeutic characteristics against oxidative stress and inflammatory damage [189,190]. The effects of GSPE on obese rats included the stimulation of energy expenditure, an increase in thermogenic capacity, and inhibiting mitochondrial dysfunction in brown adipose tissue [191]. Rats treated with GSPE had fewer mitochondrial degenerations, stabilized mitochondrial enzymes, and corrected mitochondrial dysfunction in myocardium and brown adipose tissue [191,192,193]. GSPE served to reduce proteinuria and podocyte injury as well as nephropathy progression in diabetic rats [194]. Further, the antioxidant capacity of GSPE enhanced the activity of SOD2 and CAT and decreased the levels of MDA and inflammatory cytokines, such as TNF-α and Monocyte chemoattractant protein (MCP1), in renal tissues of diabetic rats [195,196]. Additionally, GSPE was able to restore mitochondrial DNA and increase Nrf1 and TFAM RNA expression, which could suppress renal mitochondrial dysfunction [123]. In addition, GSPE protected diabetic podocytes from injury by restoring phospho-AMPK, SIRT1, and PGC-1α levels [123]. It was shown that protein SIRT1 was the therapeutic target of GSPE against H_2_O_2_ injury. GSPE upregulated the SIRT1 and re-established homeostasis of mitochondrial complexes I, II, III, and IV, enhanced antioxidant enzymes, such as SOD2, whereas it inhibited apoptosis factors, such as BAX and P53, in HEK-293 cells [197]. Further, GSPE increased GSH and TBARS and the protein levels of Nrf2, HO-1, and GST in diabetic kidney and nephrotoxicity [198,199]. By reducing ROS levels, GSPE protected kidneys from oxidative-stress-induced injury [195]. Further, GSPE inhibited NF-κB in I/R injuries in Rats; therefore, it reduced the markers of renal injury and oxidative damage and even inactivated the inflammatory pathway [200]. Thus, GSPE reduced renal damage in rats by activating the Nrf2 signaling pathway, which consequently improved the antioxidant capacity of the tissue [198]. These studies revealed that GSPE might be a safe therapeutic candidate to regulate mitochondrial dysfunction in kidney diseases.

### 5.8. Hesperetin

As a natural flavonoid found in citrus plants [201], hesperetin has antioxidant, cardiovascular regulation, and anti-cancer activities [93]. Oxidative stress and ROS generation are the significant factors in cisplatin-induced AKI [202]. Hesperetin reduces the renal MDA and NO levels and restores the antioxidant enzyme levels, such as GSH, CAT, GPx, and SOD, to the normal levels in rats with nephrotoxicity [93]. It was reported that the levels of MDA and NO in the kidneys reduced by hesperetin and the levels of antioxidant enzymes, such as GSH, CAT, GPx, and SOD, were restored to normal levels. Hesperetin significantly normalized the elevated level of inflammatory cytokines, such as TNF-α, IL-1β, and IL-6, and, thus, protected the kidney from inflammatory insult in rats with nephrotoxicity [93,203]. Moreover, hesperetin inhibited phosphorylation of Akt in diabetic nephropathy, indicating that the PI3K/Akt pathway could be involved in the protective effects of hesperetin [204]. Hesperetin also inhibited JNK, ERK, and p38 phosphorylation, suggesting that it could inhibit cisplatin-induced inflammation [205]. Activating the Nrf2 signaling pathway by hesperetin significantly diminished the oxidative damage of ARPE-19 cells and promoted the SIRT6 expression to protect from I/R injury [206,207]. It was shown that hesperetin could inhibit the apoptosis induced by cisplatin, decrease Bax and caspase-3 expression, and increase Bcl-2 expression [208]. Overall, hesperetin protects against nephrotoxicity and diabetic kidney injury by inhibiting inflammation, oxidative stress, and apoptosis.

### 5.9. Ellagic Acid

**Figure 2:**
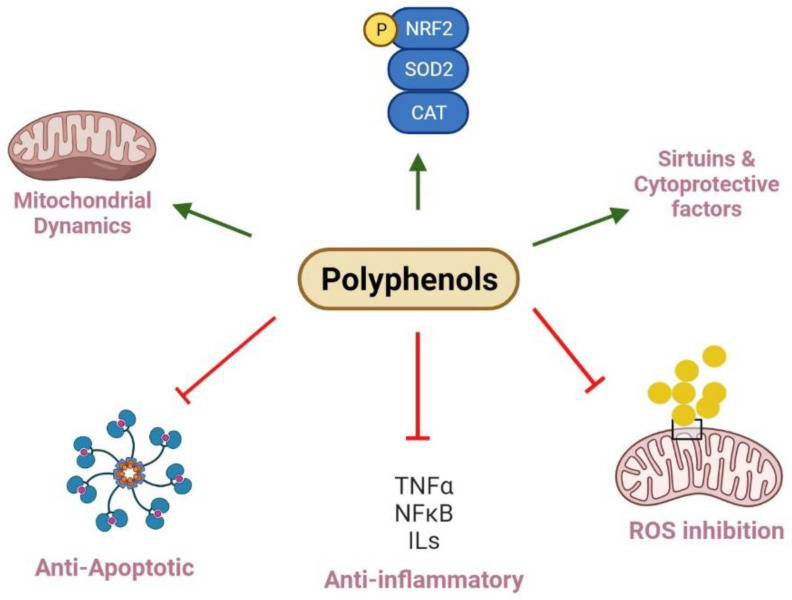
Polyphenols and their roles in renal mitochondrial dysfunction. Polyphenols regulate mitochondria biogenesis and dynamics via increasing Nrf2 and PGC-1α expression and balancing fission and fusion events, while kidney diseases result from imbalanced mitochondria dynamics and reduce biogenesis. In this pathway, deacetylation of SIRT and phosphorylation of AMPK can positively regulate biogenesis. Polyphenols can decrease the protein levels of pro-inflammatory factors, including TNF-α, IL-1β, IL-6, and NF-κB to exert anti-inflammatory effects. They also improve mitochondria function and injury through inhibition of ROS generation. Polyphenols show protective effects through inhibiting MPT pore opening, which can trigger the release of cytochrome *C* into the cytosol, swelling of mitochondria, and activating of caspase cascade; finally, they reveal anti-apoptotic impacts. Further, polyphenols restore antioxidant enzyme levels such as SOD2 and CAT to normal levels, improve antioxidant status, and scavenge free radicals.

Ellagic acid is a phenolic acid present in fruits and vegetables, such as raspberries, strawberries, walnuts, grapes, and blackcurrants [209]. The antioxidant effect of ellagic acid leads to scavenging O2^·−^, OH^−^, and lipid peroxide, therefore, inhibiting lipid peroxidation and improving the antioxidant status [210]. A study proved that ellagic acid reduced serum MDA levels and increased SOD levels, indicating that it alleviated diabetic nephropathy symptoms by reducing oxidative stress [211,212]. Ellagic acid was also reported to lower TNF-α and IL-1β levels in diabetic nephropathy and nephrotoxicity kidney injury mice, which might be mediated through NF-κB; therefore, ellagic acid could be a potent inhibitor of NF-κB activation [211,213]. Further, ellagic acid reduced the cellular membrane damage by scavenging the free radicals in rats with nephrotoxicity and nephropathy [90]. This protection was shown by covering depleted levels of SOD, GSH, CAT, and Bcl2 in the kidney, inhibiting caspase-3 activation and increasing the Bcl-2/Bax expression ratio. They found that ellagic acid significantly reduced the mitochondrial ROS content, reversed the swelling of mitochondrial kidney, and prevented loss of mitochondria membrane potential. Further, it was suggested that the anti-apoptotic effects of ellagic acid could be attributed to the upregulation of Nrf2 [90,120,214]. Additionally, Nrf2 could suppress inflammation by inhibiting TNF-α and NF-κB in diabetic nephropathy in cell lines, an animal model, or both [215]. It also activated different antioxidant enzymes, such as HO-1, NQO1, GST, and GSH [216,217]. The dysfunction of mesangial cells in diabetic nephropathy might be related to the PI3K/Akt signaling pathway activation inhibited by ellagic acid [218]. Ellagic acid treatment also triggered SIRT1 overexpression in renal tissues, which imparted renal tolerance to oxidative stress [214]. Moreover, ellagic acid-induced SIRT1 expression suppressed p53 and promoted cell survival via expression of antioxidant enzymes, such as CAT [214]. Overall, these results suggest that ellagic acid decreases renal inflammation and oxidative stress, leading to improved kidney function (Figure 2 and Table 1).

## 6. Discussion and Perspectives

As discussed above, dysfunctional mitochondrial biogenesis, dynamics, or OXPHOS is a vital underlying factor in renal mitochondrial damage [11]. Although the commonly used drugs, such as cisplatin, gentamycin, cyclosporine A, and doxorubicin, in clinical practice have anticancer, antibiotic, and anti-inflammatory effects, they have irreversible side effects on the kidney [225]. The current literature suggests that mitochondrial dysfunction adversely alters kidney function and worsens complications that may promote complex renal diseases [6]. Renal mitochondrial alterations are associated with cellular damage, oxidative stress, inflammation, and apoptosis [226]. Eventually, the disturbed renal mitochondrial homeostasis leads to CKD, AKI derived from nephrotoxicity and I/R, and nephropathy [11]. Overall, the available studies display the need to target mitochondrial dysfunctional to restore kidney function and stimulate renal repair or prevent further damage in renal tissues. Despite the fact that defective mitochondria are linked to kidney diseases, the pathogenic relationship and our knowledge of the impact of mitochondrial dysfunction in patients with kidney disease remain uncertain. In animal models of kidney damage, mitochondria-targeting therapeutics have been shown to preserve mitochondrial structures and functions [227]. Indeed, dietary antioxidants, such as vitamin C and E, polyunsaturated fatty acids (PUFA), probiotics, N-Acetylcysteine (NAC), and exercise, may be suitable therapeutics for mitochondrial oxidative damage [12,148]. Polyphenols have shown promising potential in specific kidney injuries and diseases in animal and cell studies [18,228,229,230]. These mitochondria-targeting antioxidants have been demonstrated to effectively decrease ROS accumulation, inhibit release of pro-inflammatory cytokines, and kidney injury, and favor mitochondrial biogenesis and kidney function in various renal disease models.

Primarily, the structure of polyphenols allows them to act as an antioxidant, as they are able to donate an electron and scavenge ROS to make them stable [68,133]. Moreover, recent research has revealed that polyphenols may have more specific cell signaling mechanisms than general antioxidant actions via complex mitochondria function regulation [231]. Emerging evidence indicates that polyphenols, such as resveratrol, quercetin, curcumin, EGCG, kaempferol, ellagic acid, hesperetin, and GSPE, restore mitochondrial biogenesis by stimulating PGC-1α, NRF1/2, and TFAM to improve kidney function [72,134,139,140,198,199]. On the other hand, the down-regulation of apoptotic proteins and release of cytochrome *C* by polyphenols, such as catechin, ellagic acid, hesperetin, quercetin, and EGCG, represent an anti-apoptotic mechanism and cyto-protective impacts to prevent kidney injury [90,150,182,208]. Notably, some polyphenols, including curcumin and caffeic acid, can ameliorate the MPT pore opening, consequently preserving mitochondrial integrity [126,134]. Another mitochondrial action restricted to catechin and resveratrol inhibits MMP loss and improves ATP production through mitochondria protein complexes [130,134]. Further, polyphenols, including caffeic acid, curcumin, resveratrol, catechin, EGCG, and GSPE, may directly prevent mitochondrial dysfunction in renal injuries by enhancing the activities of mitochondrial electron transport chain complexes [170,176,197]. In addition to acting as antioxidants, polyphenols’ action include direct up-regulation of antioxidant defense systems, such as SOD, CAT, GSH, and GPx, whereas they decrease MDA and the pro-inflammatory cytokines, such as IL-12 and TNF-α-modulated NF-κB [96,106,126,137,178,179]. Taken together, polyphenols can regulate the electron transport chain activity, improve oxygen consumption, maintain the mitochondrial membrane, and support ATP generation, probably by scavenging free radicals and inhibiting protein and lipid oxidation in nephrotoxicity, I/R, and nephropathy.

Although polyphenols are natural compounds and present themselves as therapeutic possibilities, more detailed studies on the dose of polyphenols for clinical intervention are recommended. Because most of the studies are based on animals and cells, thus, the safety and effectivity of polyphenols to restore kidney mitochondria should be examined in humans. Further, pre-treatment of some polyphenols, such as caffeic acid and kaempferol, diminished the treatment duration of kidney disease, particularly nephrotoxicity [68,97]. Therefore, further investigations are required to elucidate the exact effect of pre-treatment polyphenols as a prevention agent against kidney disease. It is necessary to analyze whether polyphenols alter mitochondrial dysfunction in kidney disease compared to standard medicine; hence, they can be used as an alternative treatment compared to chemical medicine with more minor side effects. Further, it is necessary to observe interactions between clinically used medicine and polyphenols to address the safety aspects of pharmacology. There is also a lack of data to present the impact of fruits, vegetables, cereals, nuts, and plant consumption on kidney health and its mitochondria function. Further, the production of foods rich in polyphenols, food fortification, and polyphenol supplementation plays a significant role in the pharmaceutical use of this strategy. Accordingly, extensive studies on the design of new dietary patterns should be carried out.

## Figures and Tables

**Table 1:** Preclinical evidence of polyphenols as a therapeutic approach for kidney disease.

Substance	Model (Animal or Cell)	Outcomes	References
CAPE (22 mg/kg and 34 mg/kg)	Wistar rat	Protected oxidative phosphorylation of kidney mitochondrial and decreased ROS production at Complex II in ischemia/reperfusion model.	[68]
CAPE (pretreated with two doses (22 mg/kg and 34 mg/kg))	Wistar rats	Ameliorated ischemia-induced renal mitochondrial injury, improved oxidative phosphorylation with complex I-dependent substrate glutamate/malate, increased mitochondria Ca^2+^ uptake, blocked ischemia-induced caspase-3 activation, and protected kidney cells from necrosis.	[69]
Curcumin (200 mg/kg)	Sprague Dawley rats	Attenuated renal fibrosis, inflammatory response, and mitochondrial dysfunction. Inhibited the PI3K/AKT/mTOR pathway. Revealed anti-fibrotic effects mediated through the regulation of autophagy and protection of mitochondrial function.	[131]
Curcumin (60 mg/kg)	db/+ mice	Protected kidneys of diabetic mice from hyperglycemia modify oxygen consumption rate and NO synthesis and increasing in TBARS levels in mitochondria.	[219]
Curcumin (Pre-treatment with 200 mg/kg)	Wistar rats	Replenished the mitochondrial lipid peroxidase levels with pre-treatment of curcumin. Restored the cisplatin-induced modulatory effects on altered enzymatic and non-enzymatic antioxidants in kidney mitochondria.	[92]
Curcumin (400 mg/kg)	Wistar rats	Decreased mitochondrial hydrogen peroxide production, increased the respiration related to oxidative phosphorylation and mitochondrial membrane potential, reduced fission and enhanced fusion, and increased the expression of the PGC1α and TFAM.	[138]
Curcumin (200 mg/kg)	Wistar rats	Prevented the increase of mitochondrial Fis1 protein, decreased OPA1 and SIRT3, and increased in the mitophagy associated proteins Parkin and PINK1.	[142]
Curcumin (400 mg/kg)	Wistar rats	Attenuated the decrease in activities of respiratory complexes I and IV and induction of calcium-dependent permeability transition in gentamycin-induced mitochondrial alterations. Mediated mitochondrial functions and biogenesis through nuclear factor Nrf2.	[134]
Curcumin (diet containing 0.04% (*w*/*w*) curcumin)	C57BL/6 mice	Exerted beneficial effects include increasing mitochondrial biogenesis, alleviating mitochondrial dysfunction by increasing ATP levels, activities of mitochondrial electron transport chain complexes and mitochondrial respiration, and suppressing mitochondrial membrane potential.	[220]
Quercetin (20 mg/kg for animal, 20 μM for cell)	Sprague Dawley rats Renal tubular epithelial cells	Enhanced mitophagy. The antifibrotic effect was through activation of SIRT1/PINK1/Parkin-mediated mitophagy.	[153]
Quercetin (10 mg/kg)	Wistar rats	Ameliorated the cytotoxic effects of doxorubicin and cyclophosphamide on the kidney through the elevation of antioxidant expression and the suppression of lipid peroxidation. Suppressed the accumulation of MDA and increased GPx levels.	[107]
Resveratrol (40 mg/kg)	C57BL/6 mice	Improved renal function and inflammation in aging mice. Increased the expression of Nrf2-HO-1-NQO1 signaling and SIRT1-AMPK-PGC1α signaling.	[157]
Resveratrol (10 mg/kg)	Mice	Decreased mitochondria ROS generation by enhancing SIRT3 within the upregulation of PGC1 α and SOD2 mitochondria gene expression. Suppressed cadmium-induced apoptosis in mice kidney.	[221]
Resveratrol (30 mg/kg)	Long-Evans rats	Restored mitochondrial respiratory capacity and decreased mitochondrial ROS and lipid peroxidation following hemorrhagic shock. Increased SIRT1, PGC1α, SOD2, and CAT expression.	[72]
Resveratrol (30 mg/kg)	Long-Evans rats	Restored mitochondrial function and reduced insulin resistance. The anti-glycemic effects of resveratrol mediated by reduced mitochondrial ROS.	[222]
Resveratrol (20 mg/kg for animal, 10 μM for cell)	Sprague-Dawley rats Mouse mesangial cell	Upregulated SIRT1 and PGC1α deacetylation contributed to the mitochondrial protective effects of resveratrol.	[34]
Resveratrol (50 mg/kg)	Sprague-Dawley rats	Restored SIRT1/3 activity, decreased acetylated SOD2 levels, ameliorated oxidative stress and mitochondrial function of renal cell.	[223]
Resveratrol (diet contained resveratrol)	White chickens	Mitigated cadmium-induced oxidative stress and restored the antioxidant enzyme activity. Enhanced the phase I and II detoxification systems to relieve oxidative damage. Ameliorated cadmium-induced mitochondria dysfunction by SIRT3 upregulation and SIRT1, PGC1α, Nrf1, and TFAM transcription restrictions. Attenuated mitochondrial fission and promoted mitochondrial fusion reversed PINK1/Parkin-mediated mitophagy initiation.	[82]
Catechin (25, 50, and 100 mg/kg)	Wistar rats	Decreased MDA, NO, and TNF-α while increased SOD and CAT. Protected the kidney against the toxic effect of cadmium through its antioxidant, anti-inflammation, and mitochondrial protection.	[224]
EGCG (100 mg/kg for animal, 10 μM for cell)	C57BL/6 mice HK-2 cells	Attenuated cisplatin-induced mitochondrial oxidative stress and mitochondrial damage to electron transport chain activities while improved antioxidant defense enzyme activities in mitochondria.	[181]
Kaempferol (200 mg/kg)	Rat	Decreased the renal expression of Bax and cleaved caspase-3 and the production of ROS, MDA, TNF-α, and IL-6. Improved GSH and SOD levels and Bcl2 mRNA. Increased renal mRNA and SIRT1 protein levels that was related to increased acetylation of Nrf2 and NF-κB.	[106]
Kaempferol (200 mg/kg)	Balb/C mice	Modulated oxidative stress, inflammation, and apoptosis via ERK and NF-κB pathway. Corrected the levels of renal antioxidants and elevated the nuclear levels of HO-1 and Nrf2 in renal tissues. Attenuated the cisplatin mediated apoptosis via down-regulating the levels of Bax/Bcl2 imbalance and activating caspase-3.	[97]
GSPE (125, 250, and 500 mg/kg)	Sprague-Dawley rats	Ameliorated podocyte injury in diabetic nephropathy by activation of AMPK-SIRT1-PGC1α signaling, inhibited oxidative stress and mitochondrial dysfunction in the kidney.	[123]
GSPE (100 μM)	HEK-293 cells	Prevented H_2_O_2_ induced oxidative damage to proteins and lipids and depletion in SOD activity. Prevented mitochondrial electron transport chain dysfunction, ATP depletion, and apoptosis induced by H_2_O_2_. Regulated SIRT 1 and 3 expressions.	[197]
GSPE (125 and 250 mg/kg)	Sprague Dawley rats	Decreased renal damage by activating the Nrf2 signaling pathway; consequently, enhanced the antioxidant capacity of the tissue in diabetic rats.	[198]
Hesperetin (2.5, 5 and 10 μM)	HK2 cells	Attenuated oxidative-stress-induced apoptosis by reducing ROS levels in cisplatin-treated HK-2 cells. Activated the Nrf2 signaling pathway and regulating its downstream genes, including NQO1 and HO-1. Attenuated the MAPK signaling pathway against inflammation and inhibited the expression of apoptotic proteins to protect kidneys from AKI caused by cisplatin.	[205]
Ellagic acid (40 µM)	MCs	Protected mesangial cells from high glucose-induced injury. Inhibited some inflammatory factors and activation of PI3K/Akt signaling pathway.	[218]
Ellagic acid (100 mg/kg)	Sprague Dawley rats	Protected gentamicin-induced mitochondrial damage by preventing MMP loss and decreased mitochondrial ROS content, mitochondrial swelling, and cytochrome *C* release.	[90]
Ellagic acid (150, 100, and 50 mg/kg for animals, 100 μg/mL for cells)	Mice NRK52E cells	Ameliorated Streptozotocin induced oxidative renal injury by inhibiting NF-κB pathway.	[211]
Ellagic acid (10 mg/kg)	Wistar rats	With antioxidant and anti-apoptotic effects through overexpression of SIRT1 in renal tissues led to the decrease in renal MDA content and P53 protein level and an increase in renal GSH level and CAT activity.	[214]

## Data Availability

Not applicable.
